# Rabson Mendenhall Syndrome caused by a novel missense mutation

**DOI:** 10.1186/s13633-016-0039-1

**Published:** 2016-11-17

**Authors:** Krishnapradeep Sinnarajah, M. B. K. C. Dayasiri, N. D. W. Dissanayake, S. T. Kudagammana, A. H. H. M. Jayaweera

**Affiliations:** 1 Teaching Hospital, Peradeniya, Sri Lanka; 2 Department of Paediatrics, University of Peradeniya, Peradeniya, Sri Lanka

**Keywords:** Insulin resistance, Acanthosis nigricans, INSR gene, Rabson-Mendenhall syndrome

## Abstract

**Background:**

Rabson Mendenhall syndrome is a rare endocrine condition characterized by severe insulin resistance and hyperglycemia. It occurs due to mutations in the insulin receptor gene. Few mutations which are associated with Rabson Mendenhall syndrome have been identified and reported in the past. The management of this condition is extremely challenging and will need multi-disciplinary approach.

**Case presentation:**

An 11 year old boy presented with polyuria and polydipsia. He was noted to have coarse facies, severe acanthosis nigricans, hypertrichosis, retarded growth and developmental delay. Investigations revealed severe hyperglycemia which was poorly responsive to high doses of insulin. A diagnosis of Rabson Mendenhall syndrome was suspected based on his physical characteristics in the presence of insulin resistance. Genetic studies revealed a homozygous missense mutation in the Insulin receptor gene confirming the diagnosis of Rabson Mendenhall syndrome. This is a novel mutation which has not been reported previously.

**Conclusion:**

Rabson Mendenhall syndrome should be suspected in a patient with characteristic physical features, severe hyperglycemia and insulin resistance. The genetic studies will not only confirm the diagnosis but also will help in counselling. Wider collaboration is needed to identify definitive treatment options for managing this rare condition.

## Background

Rabson Mendenhall syndrome (RMS) is a rare autosomal recessive disorder characterized by severe insulin resistance due to mutations in insulin receptor gene (INSR gene). It is characterized by coarse facial features, growth retardation, severe acanthosis nigricans, hypertrichosis, dental abnormalities, large external genitalia, fasting hyperglycaemia and hyperinsulinemia. The condition has been described in few isolated case reports and is due to mutation in gene locus *INSR*; 19p13.3-p13.2. We are presenting a case due to a genetic lesion hitherto not previously described. The unequivocal result of genetic testing helped us to confirm the diagnosis, counsel the parents on the management options and discuss regarding the long term prognosis. The treatment options are limited for this condition and controlling the hyperglycemia is a daunting task, hence multi-disciplinary approach is important when managing this challenging clinical condition.

## Case presentation

An 11 year old boy from a rural area in Sri Lanka presented to us with polyuria, polydipsia of one year duration and intermittent headache of one month duration.

He was the first born child to second degree consanguineous parents from a poor socio-economic background. The antenatal and the perinatal periods were uneventful. He was born at term with a birth weight of 2.5 kg(-2SD) and length of 46 cm (-2SD). His developmental milestones were delayed, and he achieved his pincer grasp at 2 years, was able to walk at 3 years and spoke his first meaningful word at 4 years.

At the age of 7 years the child had developed darkening of the skin involving the neck and axillae, which had gradually increased in severity. The parents had also noticed that this boy had excessive hair growth all over his body. Two months prior to the current presentation he had become thin and was very lethargic thus prompting parents to seek medical attention. Except for few upper respiratory tract infections he did not have any significant past medical problems. There were no hospital admissions with hypoglycemia or hyperglycemia before. There is no family history of any significant medical problems. His two younger siblings are healthy and did not have any dysmorphic features.

**Fig. 1:**
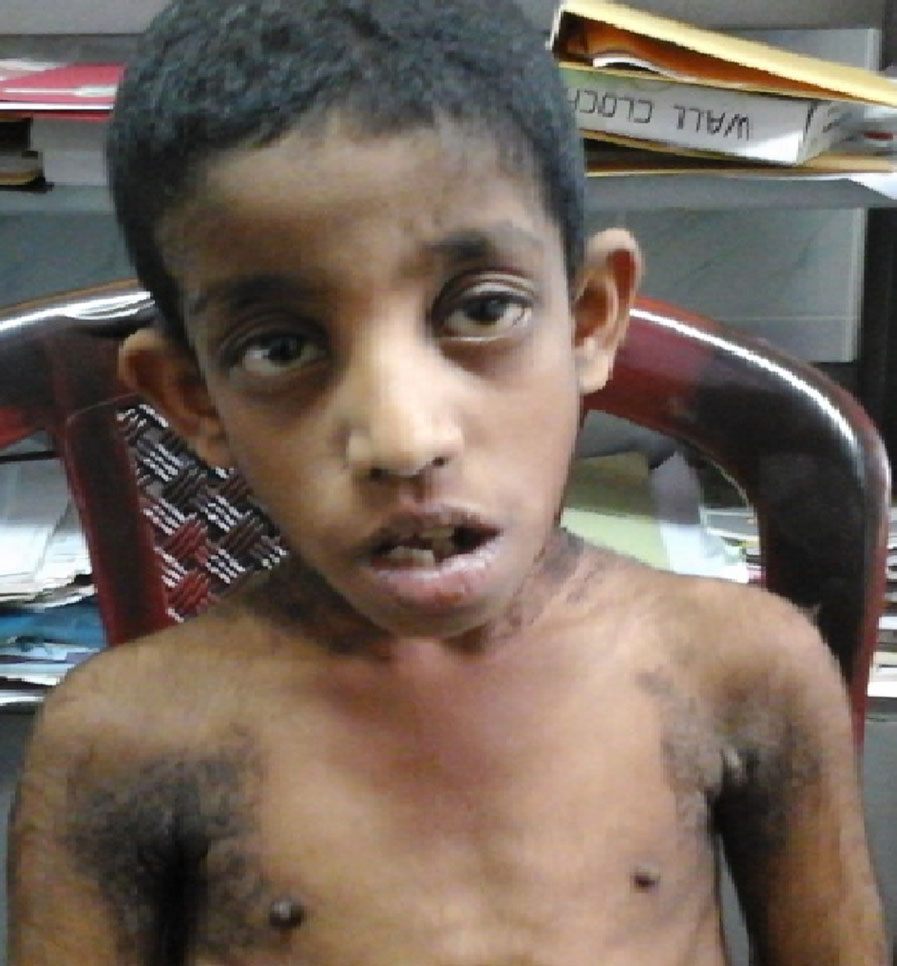
Coarse facies

**Fig. 2:**
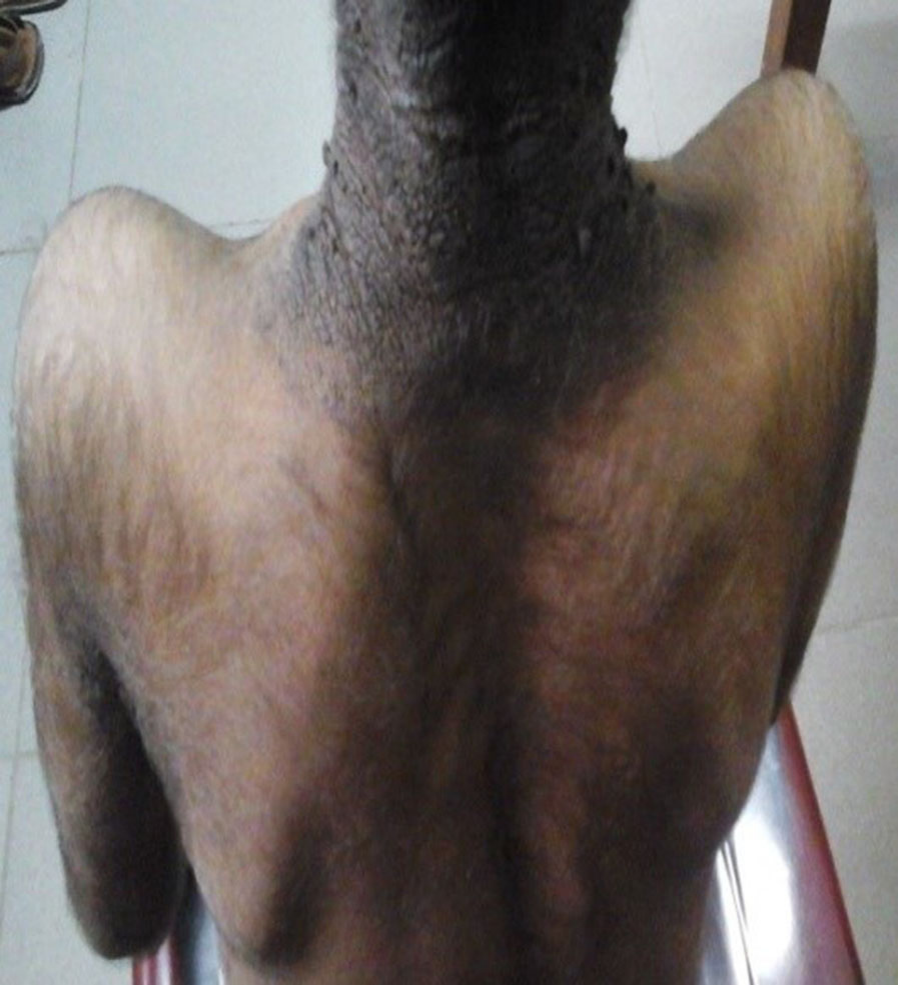
Acanthosis nigricans and hypertrichosis

On examination he weighed 17.5 kg (-3SD), his height was 111 cm (-6SD), head circumference 47 cm (-3SD), weight for height was -1SD to –2SD indicating significant growth failure. Facial dysmorphism characterized by small head, large ears, broad nose, prognathism, malocclusion of teeth were noted along with prominent nipples. There was extensive acanthosis nigricans, hypertrichosis, thick finger nails and coarse hands (Figs. 1 and 2).

Examination of the cardiovascular system, respiratory system and neurological system were apparently normal, abdominal examination did not reveal any organomegaly and his external genitalia appeared to be normal. The blood pressure was 100/60and was within the normal limits for his age. Developmental assessment showed that his motor skills and vision were age appropriate, but his speech was delayed and he could make only two word sentences. Formal assessment showed that this boy also has moderate learning difficulties.

Investigations revealed significantly raised Random blood sugar (RBS), Fasting blood sugar (FBS)and Glycated Hemoglobin (HbA1c) (Table 1). There was no associated ketonuria or ketonemia. Serum electrolytes and renal functions were within the normal range. Ultrasonography of the renal tract revealed that both his kidneys were significantly enlarged for the age. Measuring the insulin levels and C-peptide levels would have been very useful at this point, but due to the unavailability of these tests in state hospitals and the cost we could not perform the tests.

**Table 1:** The investigation results of the patient

	Results	Normal value
White blood cell count (WBC)	9.5 x 10^9^/L	4-11 x 10^9^/L
Hemoglobin	111 g/L	11.5-16 g/L
Platelet	278 x 10^9^/L	150-400 x 10^9^/L
Urea	3.5 mmol/L	1.78-8.5 mmol/L
Creatinine	60 umol/L	50-95 umol/L
Potassium	4.2 mmol/L	3.5-5.5 mmol/L
Sodium	138 mmol/L	136-145 mmol/L
Alanine Amino transferase	40 U/L	<45 U/L
Aspartate Amino transferase	26 U/L	<35 U/L
Protein -total	71 g/L	64-83 g/L
Albumin	42 g/L	35-52 g/L
Random blood sugar (RBS)	34 mmol/L	2.2-7.7 mmol/L
Fasting blood sugar (FBS)	12 mmol/L	2.2-5.49 mmol/L
HbA1c	14.2 %	Normal: Less than 5.7 % Diabetes: 6.5 % or higher
Triglycerides	1.5 mmol/L	<1.695 mmol/L
Cholesterol	4.1 mmol/L	<5.18 mmol/L
Thyroid stimulating hormone (TSH)	1.2 mIU/L	6–18 years: 0.37–6.00mIU/L

Based on the clinical findings, our patient underwent genetic studies to detect possible mutations in the INSR gene. Genomic DNA was isolated from the patient’s blood sample and enriched for the coding exons of targeted genes using hybrid capture technology. Prepared DNA libraries were then sequenced using a next generation sequencing technology at Fulgent Diagnostics, California, USA. Variants were interpreted manually using locus specific databases, literature searches, and other molecular biological principles. A homozygous variant in the INSR gene, NM_000208.2:c.1042A > T (p. Ile348Phe), was identified in our patients’ sample. This particular missense mutation has not been reported previously in patients with RMS. Polyphen2 online tool was used to assess the effect of this novel missense mutation and it was stated as possibly damaging.

After the initial assessment the boy was started on a combination of Soluble and Isophane insulin 0.5U/kg/day and then increased to 1U/kg/day. As the blood sugars were still high, Metformin was added to counter the insulin resistance. Subsequently the dose of Insulin was further increased to achieve normal glycemic control. With a Metformin dose of 500 mg three times a day and Insulin dose of 2U/kg/day the blood sugar levels were maintained below 15 mmol/L. After starting the treatment in order to titrate the Insulin doses this boy underwent blood sugar series test which showed that the pre-prandial sugar levels were in the in the range of 10-12 mmol/L and post prandial blood sugar levels were 12-14 mmol/L. Due to the financial constraints on the family, expensive newer diabetes drugs were not considered.

Referrals to the Dermatologist, Orthodontist and Endocrinologist were done for management of other associated problems. Parents were counselled about the course of the disease, management options, long term prognosis and recurrence in the family. The counselling was very useful to alleviate the psychological stress of the parents and to face the challenges in dealing with a child with RMS. None of the immediate family members of the index patient had any physical features or biochemical abnormalities suggestive of Rabson Mendenhall syndrome. Unfortunately, due to the high cost of the test, parents and the siblings did not undergo the genetic analysis.

In our patient the diagnosis of RMS was made based on the clinical presentation with significant acanthosis nigricans, hirsuitism, dysmorphic characteristics, growth retardation, insulin dependent diabetes requiring large amounts of insulin and this was confirmed by genetic analysis.

## Discussion

The need for relatively higher dose of insulin to manage the hyperglycaemia suggested severe insulin resistance in our patient. Insulin resistance is defined as “a state of a tissue in which a greater than normal amount of insulin is required to elicit a quantitatively normal response” [1]. Synthesis of insulin receptors is regulated by INSR gene, hence mutations in these genes will lead to insulin resistance.

Insulin receptors are heterotetrameric receptors consisting of two (α) and two (β) subunits. Insulin binds to the extracellular subunits of its receptor and activates the intracellular tyrosine kinase in the transmembrane subunits [2]. Tyrosine kinase phosphorylates and recruits certain intracellular intermediates such as insulin-like substrates 1 and 2, Shc, and Gab1 [3]. These intermediate substances activate other signalling molecules like Growth factor receptor-bound protein 2, Cytoplasmic protein Nck, tyrosine phosphatase and the phosphoinositide 3-kinase, amplifying the initial effect generated by the insulin binding to its receptor. These complex interactions finally produce the expected insulin effects like uptake of glucose and amino acid by the cells, glycogenesis, fatty acid synthesis and cholesterol synthesis. In addition, insulin inhibits gluconeogenesis in the liver [2]. Any defects at any of the above mentioned steps of this complex pathway can lead to insulin resistance.

Many syndromes and conditions are currently identified with insulin resistance. The common clinical conditions with insulin resistance due to receptor problems are Type A insulin resistance syndrome, HAIR-AN syndrome, Donohue syndrome and Rabson Mendenhall syndrome (RMS) [1, 4]. The pathophysiology of RMS is explained by abnormality of the INSR gene at the gene map locus 19p13.2. (OMIM: 609968, 246200, 262190) resulting in severe biallelic loss-of-function of the insulin receptor and impaired insulin binding [3, 5].

The RMS was first described by Rabson and Mendenhall in 1956.They described three siblings with Insulin-resistant diabetes mellitus having characteristic facial, skin, skeletal and dental features [6]. RMS is characterized by growth retardation, coarse facies, and skin manifestations such as hyperpigmentation, acanthosis nigricans and hypertrichosis. Dental abnormalities, enlarged phallus in males, clitoromegaly in females, nephromegaly, nephrocalcinosis, paradoxical fasting hypoglycemia, postprandial hyperglycemia, severe insulin resistance and hyperinsulinemia are other associated findings. [7] The paradoxical fasting hypoglycaemia is explained by inappropriately elevated insulin levels during fasting due to the excessive production of insulin and the prolonged half-life of the hormone signalling via the Insulin-like growth factor type 1 (IGF-1) receptor [7]. With time these patients progress to persistent hyperglycaemia and diabetic ketoacidosis. This is due to progressive decline of insulin levels, which is inadequate to prevent glucose synthesis in the liver and prevent release of fatty acid by adipocytes (7). Acanthosis nigricans, hypertrichosis and nephromegaly is too believed to be due to aberrant insulin signalling on skin and other tissues mediated through activation of IGF-1 receptor [8].

Although RMS and Donohue syndrome have many similarities, RMS is distinguished by the presence of less severe clinical symptoms, more severe coarse features, dysplastic dentition, gingival hyperplasia, pineal gland hyperplasia, diabetic ketoacidosis and survival beyond infancy. Donohue syndrome is characterized by unique facial features like globular eyes, micrognathia, severe intra uterine growth retardation, lipoatrophy, early onset severe fasting hypoglycemia and early infantile death [9–11].

Our patient had most of the clinical features suggestive of RMS. Interestingly he did not have some features such as phallic enlargement, nephrocalcinosis and fasting hypoglycemia. Absence of fasting hypoglycemia can be expected in this age because as the disease progresses children with RMS tend to develop persistent hyperglycemia [7]. For confirmation of the diagnosis of RMS, he underwent genetic analysis which revealed a novel homozygous variant in the INSR gene, NM_000208.2:c.1042A > T (p. Ile348Phe). Various INSR mutations have been reported previously in patients with RMS (Table 2) [12]. An exhaustive literature survey revealed that this will be the first genetically confirmed published case of RMS with the genetic mutation p. Ile348Phe. A similar amino acid conversion was observed in another reported case of RMS, but situated 27 amino acids away from the mutation reported in our patient, hence the mutation in our patient is unique and novel [12].

**Table 2:** Previously reported INSR mutations associated with Rabson–Mendenhall syndrome [5]

No	INSR mutation
1	Pro193Leu Pro193Leu
2	Cys284Tyr Cys284Tyr
3	Ser323Leu Ser323Leu
4	Ile1116Thr Arg1131Trp
5	Pro970Thr Arg1131Trp
6	Arg1174Trp WT
7	Asn878Ser Ala1162Val
8	Cys159Phe Arg229Cys
9	Asn15Lys Arg1000X
10	Arg209His Gly359Ser
11	Arg86X Mu p.Asp261_Leu262insLeuHisLeuVal
12	IVS4-2A > G c.2480-2487del
13	Ile321Phe

Several therapeutic options are currently available for managing children with RMS. Metformin and Pioglitazone are effective in the initial period of the management [13]. Exogenous insulin in high doses can be helpful when there is uncontrolled hyperglycemia and ketoacidosis, but in long run usually fails to provide adequate glycaemic control [1, 14]. New treatment modalities like recombinant IGF-1 and subcutaneous Leptin have shown promising results, but those have been tried only in a few cases [15]. But despite all these measures, the long term prognosis of patients with RMS remains poor until now. Hence we believe that depth research and international collaboration is required to identify new therapeutic options for children with this challenging condition.

## Conclusion

Rabson Mendenhall can be clinically suspected based on characteristic physical features and metabolic derangements. But genetic studies should be done to confirm the diagnosis. Although the developing countries lack the genetic testing facilities the genetic results will undoubtedly help to identify the mutation, discuss about the nature of the disease, management options, long term consequences and predict the recurrence in the family. It will also facilitate to screen the asymptomatic immediate family members. So all efforts should be made to develop cost effective genetic and molecular testing in developing countries. Wider collaboration is needed to share the new knowledge about the treatment modalities available for managing this unique and difficult condition.

## Abbreviations

FBS: Fasting blood sugar
HAIR-AN syndrome: Hyper androgenism, Insulin resistance, Acanthosis nigricans syndrome
HbA1c: Glycated Hemoglobin A1c
INSR gene: Insulin receptor gene
RBS: Random blood sugar
RMS: Rabson Mendenhall syndrome
TSH: Thyroid stimulating hormone
